# Structure Identification and Functional Mechanism of Natural Active Components: A Special Issue

**DOI:** 10.3390/foods11091285

**Published:** 2022-04-28

**Authors:** Zhaohua Huang, Miao Zhu, Guowen Zhang

**Affiliations:** State Key Laboratory of Food Science and Technology, Nanchang University, Nanjing East Road 235, Nanchang 330047, China; zhaohuahuang@email.ncu.edu.cn (Z.H.); 13617912638@163.com (M.Z.)

The natural active components derived from plants have attracted widespread attention due to their abundant species and source advantages. With the continuous deepening of research, studies have shown that many natural active components have broad-spectrum biological activities, such as antioxidant, antihypertensive, hypoglycemic, anti-inflammatory, antibacterial, anticancer, and enzyme-inhibiting activity properties, which are valuable sources of research and development in functional food factors and novel drugs. Systematical studies on the structure of components, physiological activities, the structure–activity relationship, and mechanisms of action for active components using modern scientific methods and experimental means are hot research topics. In addition, the exploration of the combined effect and mechanism of various natural bioactive substances will provide a theoretical basis for the further processing and comprehensive development of resources at multiple levels and from various points of view. This Special Issue of *Foods*, entitled “Structure Identification and Functional Mechanism of Natural Active Components”, provides a forum for researchers to communicate some of their latest findings in this field. Subsequent to the peer review process, 9 original research articles were included in this Special Issue of *Foods*.

Tang et al. [1] used the natural ingredient stigmasterol as an oleogelator to explore the effect of concentration on the properties of organogels. Organogels based on rapeseed oil were investigated using various techniques (oil binding capacity, rheology, polarized light microscopy, X-ray diffraction, and Fourier transform infrared spectroscopy) to better understand their physical and microscopic properties. Results showed that stigmasterol was an efficient and thermoreversible oleogelator, which is capable of structuring rapeseed oil at a stigmasterol concentration as low as 2% with a gelation temperature of 5 °C. The oil binding capacity values of organogels increased to 99.74% as the concentration of stigmasterol was increased to 6%. The rheological properties revealed that organogels prepared with stigmasterol formed a pseudoplastic fluid with non-covalent physical crosslinking, and the G’ of the organogels did not change as the frequency of scanning increased, showing its characteristic of a strong gel. The microscopic properties and Fourier transform infrared spectroscopy showed that stigmasterol formed rod-like crystals through the self-assembly of intermolecular hydrogen bonds, fixing rapeseed oil in its three-dimensional structure to form organogels. Therefore, stigmasterol can be considered as a good organogelator. It is expected to be widely used in food, medicine, and other biological-related fields.

*Lactiplantibacillus plantarum* could regulate certain physiological functions through the AI-2/LuxS-mediated quorum sensing (QS) system. Qian et al. [2] explored the regulation mechanism on the growth characteristics and bacteriostatic ability of *L. plantarum* SS-128. In their work, a *luxS* mutant was constructed using a two-step homologous recombination. Compared with Δ*luxS*/SS-128, the metabolites of SS-128 had stronger bacteriostatic ability. The combined analysis of transcriptomics and metabolomics data showed that SS-128 exhibited higher pyruvate metabolic efficiency and energy input, followed by a higher LDH level and metabolite overflow compared to Δ*luxS*/SS-128, resulting in stronger bacteriostatic ability. The absence of *luxS* induces a regulatory pathway that burdens the cysteine cycle by quantitatively drawing off central metabolic intermediaries. To accommodate this mutations, Δ*luxS*/SS-128 exhibited lower metabolite overflow and abnormal proliferation. These results demonstrate that the growth characteristic and metabolism of *L. plantarum* SS-128 are mediated by the AI-2/LuxS QS system, which is a positive regulator involved in food safety. It would be helpful to further investigate the bio-preservation control potential of *L. plantarum*, especially when applied in food industrial biotechnology.

Alzheimer’s disease (AD) is one of the most prevalent chronic neurodegenerative diseases in elderly individuals, which can cause dementia. Acetylcholinesterase (AChE) is regarded as one of the most popular drug targets for AD. Herbal secondary metabolites are frequently cited as a major source of AChE inhibitors. In the study of Liao et al. [3], baicalein, a typical bioactive flavonoid, was found to inhibit AChE competitively, with an associated IC_50_ value of 6.42 ± 0.07 µM through a monophasic kinetic process. AChE fluorescence quenching via baicalein was a static process. The binding constant between baicalein and AChE was an order of magnitude of 10^4^ L mol^−1^, and hydrogen bonding and hydrophobic interaction were the major forces in forming the baicalein−AChE complex. Circular dichroism analysis revealed that baicalein caused the AChE structure to shrink and increased its surface hydrophobicity by increasing the α-helix and β-turn contents and decreasing the β-sheet and random coil structure contents. Molecular docking revealed that baicalein predominated at the active site of AChE, likely tightening the gorge entrance and preventing the substrate from entering and binding with the enzyme, resulting in AChE inhibition. The preceding findings were confirmed by molecular dynamics simulation. The current study provides an insight into the molecular-level mechanism of baicalein interaction with AChE, which may offer new ideas for the research and development of anti-AD functional foods and drugs.

Rosemary (*Rosmarinus officinalis* L.) represents a medicinal plant known for its various health-promoting properties. Its extracts and essential oils exhibit antioxidative, anti-inflammatory, anticarcinogenic, and antimicrobial activities. The main compounds responsible for these effects are diterpenes carnosic acid, carnosol, and rosmanol, as well as the phenolic acid ester rosmarinic acid. However, surprisingly, little is known about the molecular mechanisms responsible for the pharmacological activities of rosemary and its compounds. To discern these mechanisms, Lešnik and Bren performed a large-scale inverse molecular docking study to identify their potential protein targets [4]. Listed compounds were separately docked into the predicted binding sites of all non-redundant holo proteins from the Protein Data Bank, and those with the top scores were further examined. Lešnik and Bren focused on proteins directly related to human health, including human and mammalian proteins, as well as proteins from pathogenic bacteria, viruses, and parasites. The observed interactions of rosemary compounds indeed confirm the aforementioned activities, whereas the authors also identified their potential for anticoagulant and antiparasitic actions. The obtained results were carefully checked against the existing experimental findings from the scientific literature, and as further validated using both redocking procedures and retrospective metrics.

Taking into consideration the importance of biofilms in food deterioration and the potential risks of antiseptic compounds, antimicrobial agents derived from natural products are a more acceptable choice for preventing biofilm formation and in attempts to improve antibacterial effects and efficacy. Citrus flavonoids possess a variety of biological activities, including antimicrobial properties. Therefore, in the study of Wen et al. [5], the anti-biofilm formation properties of the citrus flavonoid naringenin on the *Staphylococcus aureus* ATCC 6538 (*S. aureus*) were investigated using subminimum inhibitory concentrations (sub-MICs) of 5~60 mg/L. The results were confirmed using laser and scanning electron microscopy techniques, which revealed that the thick coating of *S. aureus* biofilms became thinner and finally separated into individual colonies when exposed to naringenin. The decreased biofilm formation of *S. aureus* cells may be due to a decrease in cell surface hydrophobicity and exopolysaccharide production, which is involved in the adherence or maturation of biofilms. Moreover, transcriptional results show that there was a downregulation in the expression of biofilm-related genes and alternative sigma factor *sigB* induced by naringenin. This work provides insight into the anti-biofilm mechanism of naringenin in *S. aureus* and suggests the possibility of naringenin use in the industrial food industry for the prevention of biofilm formation.

*Saussurea involucrate* (*S. involucrata*) was reported to have an anti-hepatoma function, but the mechanism is complex and unclear. To evaluate the anti-hepatoma mechanism of *S. involucrate* comprehensively and make a theoretical basis for the mechanical verification of later research, in the study of Gao et al. [6], the total phenolic acids from *S. involucrate* determined by a cell suspension culture (ESPI) was mainly composed of 4,5-dicaffeoylquinic acid, according to LC–MS analysis. BALB/c nude female mice were injected with HepG2 cells to establish an animal model of a liver tumor before being divided into a control group, a low-dose group, a middle-dose group, a high-dose group, and a DDP group. Subsequently, EPSI was used as the intervention drug for mice. Biochemical indicators and differences in protein expression determined by TMT quantitative proteomics were used to resolve the mechanism after the low- (100 mg/kg), middle- (200 mg/kg), and high-dose (400 mg/kg) interventions for 24 days. The results showed that EPSI can not only limit the growth of HepG2 cells in vitro, but can also inhibit liver tumors significantly, with no toxicity at high doses in vivo. Proteomics analysis revealed that the upregulated differentially expressed proteins (DE proteins) in the high-dose group were over three times that in the control group. ESPI affected the pathways significantly associated with the protein metabolic process, metabolic process, catalytic activity, hydrolase activity, proteolysis, endopeptidase activity, serine-type endopeptidase activity, etc. The treatment group showed significant differences in the pathways associated with the renin-angiotensin system, hematopoietic cell lineage, etc. In conclusion, ESPI has a significant anti-hepatoma effect, and the potential mechanism was revealed.

*Tetrastigma hemsleyanum* Diels et Gilg is a herbaceous perennial species distributed mainly in southern China. The *Tetrastigma hemsleyanum* root (THR) has been prevalently consumed as a functional tea or dietary supplement. In the study of Sun et al. [7], the digestion models in vitro including colonic fermentation were built to evaluate the release and stability of THR phenolics with the methods of HPLC–QqQ–MS/MS and UPLC–Qtof–MS/MS. From the oral cavity, the contents of total phenolic and flavonoid began to degrade. Quercetin-3-rutinoside, quercetin-3-glucoside, kaempferol-3-rutinoside, and kaempferol-3-glucoside were metabolized as major components, and they were absorbed in the form of glycosides for hepatic metabolism. On the other hand, the total antioxidant capacity (T-AOC), superoxide dismutase (SOD), glutathione peroxidase (GSH-Px) activity, and glutathione (GSH) content were significantly increased, while the malondialdehyde (MDA) content was decreased in the plasma and tissues of rats treated with THR extract in the oxidative stress model. These results indicated that the THR extract is a good antioxidant substance and has good bioavailability, which can effectively prevent some chronic diseases caused by oxidative stress. It also provides a basis for the effectiveness of THR as a traditional functional food.

4-hydroxyderricin (4-HD), as a natural flavonoid compound derived from *Angelica keiskei*, has largely unknown inhibition and mechanisms in liver cancer. Gao et al. [8] investigated the inhibitory effects of 4-HD on hepatocellular carcinoma (HCC) cells and clarified the potential mechanisms by exploring apoptosis and cell cycle arrest mediated via the PI3K/AKT/mTOR signaling pathway. The results showed that 4-HD treatment dramatically decreased the survival rate and activities of HepG2 and Huh7 cells. The protein expressions of apoptosis-related genes significantly increased, while those related to the cell cycle were decreased by 4-HD. 4-HD also downregulated PI3K, p-PI3K, p-AKT, and p-mTOR protein expression. Moreover, PI3K inhibitor (LY294002) enhanced the promoting effect of 4-HD on apoptosis and cell cycle arrest in HCC cells. Consequently, the authors demonstrated that 4-HD can suppress the proliferation of HCC cells by promoting PI3K/AKT/mTOR signaling pathway-mediated apoptosis and cell cycle arrest.

Guo et al. [9] used eight extraction technologies to extract sweet tea (*Lithocarpus litseifolius* (Hance) Chun) crude polysaccharides (STPs), and investigated and compared their chemical, structural, and biological properties. The results revealed that the compositions, structures, and biological properties of STPs varied based on different extraction technologies. Protein-bound polysaccharides and some hemicellulose could be extracted from sweet tea with diluted alkali solution. STPs extracted by deep-eutectic solvents and diluted alkali solution exhibited the most favorable biological properties. Moreover, according to the heat map, total phenolic content was the most strongly correlated with biological properties, indicating that the presence of phenolic compounds in STPs might be the main contributor to their biological properties. To the best of the authors’ knowledge, this study reports the chemical, structural, and biological properties of STPs, and the results contribute to understanding the relationship between the chemical composition and biological properties of STPs.

In summary, the findings published in this Special Issue clearly indicate both the breadth and depth of the recent studies on the functional properties of natural active components. However, our understanding of natural active components is still far from adequate, and subsequent research must continue to build on previous studies. Finally, we thank the authors for their valuable contributions to this Special Issue.
